# Seroprevalence of Equine Influenza and Its Associated Risk Factors in Northwest Nigeria

**DOI:** 10.3390/pathogens11111372

**Published:** 2022-11-17

**Authors:** Olaolu T. Olufemi, Emmanuel R. Edeh, Mustapha S. Isyaku, Mustapha Haliru, Shafiu Samaila, Philip W. Mshelia, Olajide A. Owolodun, J. Richard Newton, Janet M. Daly

**Affiliations:** 1 One Virology, Wolfson Centre for Global Virus Research, School of Veterinary Medicine and Science, University of Nottingham, Loughborough LE12 5RD, UK; 2 Department of Veterinary Public Health and Preventive Medicine, Faculty of Veterinary Medicine, University of Jos, Jos 930003, Nigeria; 3 Department of Veterinary Medicine, Surgery and Radiology, Faculty of Veterinary Medicine, University of Jos, Jos 930003, Nigeria; 4 Faculty of Veterinary Medicine, Bayero University Kano, Kano 700006, Nigeria; 5 Ministry of Animal Health and Fisheries Development, Usman Faruk Secretariat, Sokoto 840103, Nigeria; 6 Department of Veterinary Medicine, Faculty of Veterinary Medicine, Ahmadu Bello University, Zaria 810107, Nigeria; 7 Biotechnology Centre, National Veterinary Research Institute, Vom 930103, Nigeria; 8 Department of Veterinary Medicine, University of Cambridge, Cambridge CB3 0ES, UK

**Keywords:** equine influenza, seroprevalence, Nigeria

## Abstract

Equine influenza (EI) is a fast-spreading respiratory disease of equids caused by equine influenza A virus (EIV), often resulting in high morbidity and a huge economic impact on the equine industry globally. In this cross-sectional study to determine the seroprevalence of EI and its associated risk factors, sera from 830 horses bled on a single occasion in Northwest Nigeria between October 2019 and January 2020 were screened for antibodies to A/equine/Richmond/1/2007 (H3N8) using the single radial haemolysis (SRH) assay. Antibodies were detected in 71.3% (592/830, 95% CI: 68–74%) of horses (SRH area ≥ 0.5 mm^2^). Although there were statistically significant univariable associations between seropositivity and age, sex, breed, purpose and coat colour, only age remained significant when included with each of the other variables in bivariable analyses. There was a clear trend for increasing odds of seropositivity with increasing age: OR 1.6, 95% CI: 1.05–2.40 (*p* = 0.03) for 5–14-year-olds and OR 8.13, 95% CI: 2.75–24.1 (*p* < 0.001) for ≥15-year-olds compared to horses <5 years old. The mean SRH value was 78.2 mm^2^ (median = 88 mm^2^, interquartile range = 0–121 mm^2^) with only 9% of the horses having an SRH value > 150 mm^2^, considered sufficient to protect against clinical disease and virus shedding. Comparative screening of a subset of the horses (n = 118) with a 2019 H3N8 virus (A/equine/Worcestershire/2019) revealed a significantly greater seropositivity (*p* = 0.0001) than A/equine/Richmond/1/2007 consistent with exposure of the population during a widespread outbreak of EI in the region in 2019. In conclusion, there was an insufficient level of protection against EI in the region and introduction of a vaccination programme with vaccines containing recently circulating virus is recommended to mitigate against further outbreaks of EI in Nigeria.

## 1. Introduction

Equine influenza (EI) is a rapidly spreading, emerging and re-emerging respiratory disease of equids [1] caused by equine influenza virus (EIV), a member of the genus *Alphainfluenzavirus* (formerly Influenza A) within the family Orthomyxoviridae [2]. It is globally recognised as causing high morbidity in susceptible equids during outbreaks, resulting in a huge economic impact on the equine industry. Two subtypes of influenza A virus have circulated in horses, H7N7 and H3N8, which were first isolated in 1956 and 1963 from horses in Czechoslovakia (A/equine/Prague/56) and the USA (A/equine/Miami/63), respectively [3,4]. Given that the last confirmed outbreak caused by the H7N7 subtype occurred in 1979, it is now believed to be extinct in horses or circulating at low levels in limited geographical regions [5,6,7,8,9]. In contrast, since 1980, to date, there have been recurring EI outbreaks caused by the H3N8 subtype, which has become an almost global threat to horses, especially in regions without routine immunization [10,11].

The equine H3N8 subtype started as a discrete genetic and antigenic lineage until divergent evolution (in around 1989) produced two distinct lineages: American and Eurasian (Europe and Asia) [12]. A further divergence of the American lineage produced the Florida and Kentucky sub-lineages [13]. The Florida sub-lineage, which diverged into two clades: Florida clade 1 (FC1) and Florida clade 2 (FC2), represented by A/equine/South Africa/4/2003-like or A/equine/Ohio/2003-like and A/equine/Richmond/1/07-like viruses, respectively, has been responsible for EI outbreaks across the globe [14]. Until recently, FC1 viruses predominated in the Americas and FC2 viruses in Europe. In 2018/2019, widespread outbreaks were caused by FC1 viruses first identified in South America [15] and subsequently in European countries [16].

In Nigeria, the first outbreak of EI was described over three decades ago among 253 horses that converged (from different parts of the country) for a polo tournament in western Nigeria [17]. The isolates from this outbreak corresponded to the strains causing disease in Europe at the time [18]. The widespread EI outbreaks caused by FC1 viruses in 2018/2019 also affected Nigeria [19] and some other countries in West and Central Africa [20]. There have been few serological reports of EI among equids in different parts of Nigeria and those that exist are focussed on stabled horses [21,22,23,24]. Most of these reports employed competition ELISA and haemagglutination inhibition (HI) tests. Antibodies against EIV can also be detected using the single radial haemolysis (SRH) assay, which offers a reproducible, reliable and continuous measure of antibody that correlates with protection level to EI. In experimental infection of vaccinated ponies, SRH values above 154 mm^2^ conferred complete protection (no clinical signs or virus shedding) against related EI strains while animals with SRH values greater than 85 mm^2^ did not exhibit clinical disease but still shed virus [25]. Findings from field studies of outbreaks confirmed 150 mm^2^ as the limit above which protection against a related strain of EIV is provided by a vaccine [26,27].

There are about 1.1 million equids in Nigeria (around 200,000 horses and 900,000 donkeys) [28] but there is a paucity of information on the seroprevalence and geographic spread of EI. The 2018/2019 outbreak, which majorly involved equids in Northwest Nigeria geopolitical zone, highlighted the need to investigate antibody levels of horses in the region to inform public health plans for the prevention and control of future outbreaks was recognised. Thus, this study investigated the seroprevalence of equine influenza in 830 horses in four of the Northwest Nigerian states and the risk factors associated with SRH seropositivity.

## 2. Materials and Methods

### 2.1. Ethical Statement

This study received approval (reference 2635181112) from the School of Veterinary Medicine and Science Ethics Clinical Review Panel, University of Nottingham, United Kingdom. 

### 2.2. Sampling

**Figure 1:**
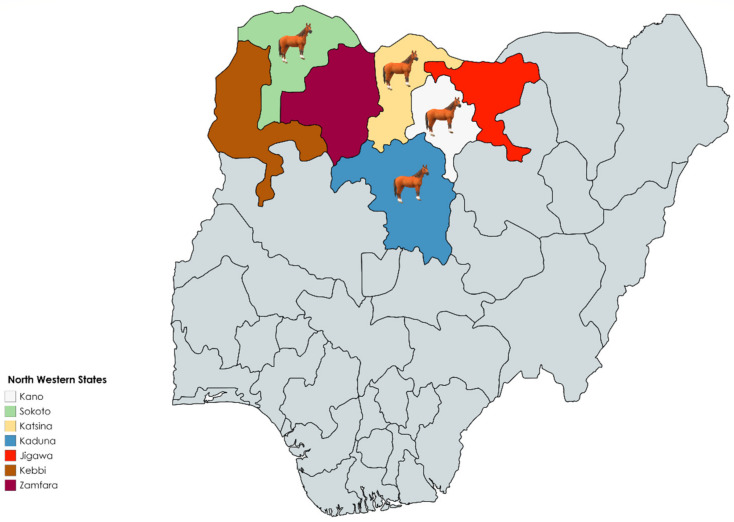
Map showing the seven states that comprise the geopolitical zone of Northwest Nigeria. Samples for this study were obtained from horses in the states of Kano, Sokoto, Katsina and Kaduna. Created using https://mapchart.net//africa-detailed.html (accessed on 25 May 2022).

In this cross-sectional study, blood samples were obtained on a single occasion from apparently healthy horses in four states (Kaduna, Kano, Katsina and Sokoto) in Northwest Nigeria (Figure 1). Horses were sampled based on availability and willingness of horse owners to participate in the study, and all horses included in this study were sampled with consent from relevant authorities and community inhabitants. The horses belonged to farms, individual horse owners, monarchs and their traditional rulers. The samples were collected from October 2019 to January 2020 during different visits to the four states. In total, 830 horses were aseptically bled by jugular venipuncture, and sera were harvested then heat inactivated before shipping to the United Kingdom. Basic information (age, breed, coat colour, location, use, sex and vaccination status) was recorded for each animal at the time of sampling. Information on influenza vaccination status was inconsistent and unreliable, especially among non-stabled horses; therefore, this was not employed in further analyses. 

### 2.3. Single Radial Haemolysis (SRH)

Single radial haemolysis (SRH) assay is an immunodiffusion assay based on virus-coupled erythrocytes. Influenza A/equine/Richmond/1/2007 (H3N8), representative of FC2, was kindly provided by Dr Debra Elton (Animal Health Trust) and A/equine/Worcestershire/2019 (H3N8), representative of FC1, by Dr Neil Bryant (Cambridge University). The viruses were propagated in embryonated hens’ eggs and antibodies to both viruses were assessed by performing the SRH assay as prescribed in the World Organisation for Animal Health (WOAH) Manual of Diagnostic Tests and Vaccines for Terrestrial Animals [29]. Digital callipers were used to measure the diameter of the zones of lysis before calculating the area (mm^2^). Serum from a hyperimmune pony was used as a positive control on every plate.

### 2.4. Statistical Analysis

Overall seroprevalence was estimated by dividing the number of seropositive horses by the total number of horses sampled. Pearson’s chi-square test was used to determine the association between explanatory variables and seropositivity to EI. The variables were categorized as follows:

States: Kaduna, Kano, Katsina and Sokoto
Age: “young” (<5 years); “adult” (5–14 years); “old” (≥15 years)
Breed: “exotic” (Argentine/Sudan/South African); “mixed (Talon); “local” (West African Dongola/Arewa)
Coat colour: “basic” (bay/chestnut/black/brown); “bright” (white/golden/pinto/roan); grey
Use: polo, durbar festival and mixed (more than one use including racing)
Sex: male; female.

Ordinary logistic regression was used to determine the risk factors predictive of EI seropositivity (the dependent variable). Initially, Pearson’s chi-square test was used on cross-tabulated data to examine the association between explanatory variables and seropositivity to EI. Ordinary logistic regression was used to determine the risk factors predictive of EI seropositivity (the dependent variable) with odds ratios (OR) and corresponding 95% confidence intervals (CI) calculated for each variable in univariable analyses. All non-collinear variables with *p* ≤ 0.2 were selected for multivariable analyses by ordinary logistic regression conducted using a forward-stepwise approach, with improvement in model fit with the addition of further variables assessed using likelihood ratio tests, with significance for variable retention set at *p* ≤ 0.05. Paired *t*-test was used to compare mean differences between paired SRH values obtained using the viruses isolated in 2007 or 2019. All analyses were performed using StataSE (17.1, StataCorp LLC, College Station, TX, USA).

## 3. Results

### 3.1. Demographic Characteristics of Horses Sampled in Northwest Nigeria

**Figure 2:**
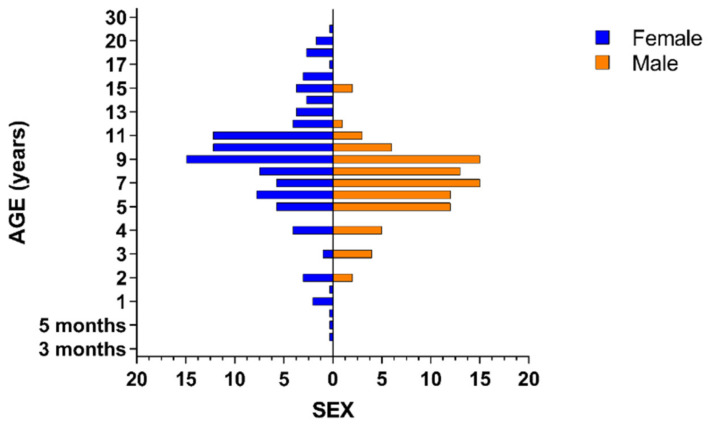
Population pyramid of raw age data versus sex.

In total, 830 horses were sampled from Kaduna (n = 256), Kano (n = 314), Katsina (n = 122) and Sokoto (n = 138). Age of horses ranged from <3 months to 30 years, with a mean age ± SD of 8.05 ± 3.75 years. The ages of the 10 horses older than 18 years were estimated rounded to the nearest 5 years, i.e., 20 (n = 5), 25 (n = 3) and 30 (n = 2). In total, 535 (64.5%) of the horses were male and 295 (35.5%) were female. Most male horses were aged between 5 and 11 years and all horses aged <2 years and >15 years were female (Figure 2). Exotic (32.3%), local (38.7%) and mixed (29%) breeds of horses having basic (77.1%), bright (9.6%) and grey (13.3%) coat colours were sampled (Table 1).

### 3.2. Prevalence of Antibodies against Influenza A/equine/Richmond/1/2007 (H3N8) among Horses Sampled in Northwest Nigeria

**Figure 3:**
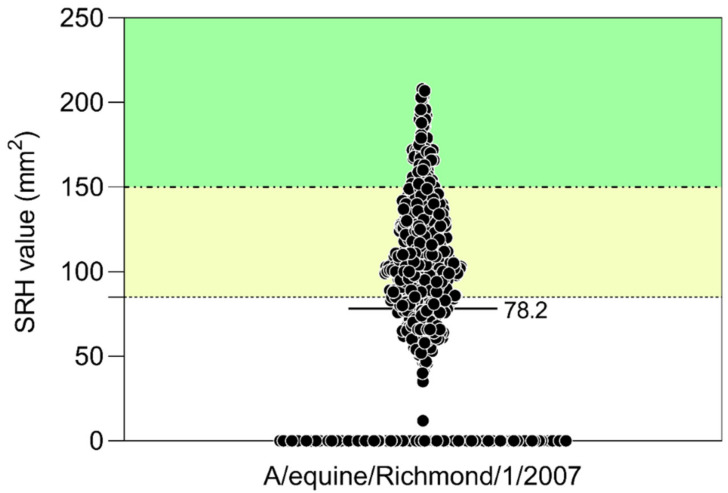
Single radial haemolysis (SRH) antibody values measured against A/equine/Richmond/1/2007 among 830 horses in Northwest Nigeria. Dashed lines and shading indicate cut-off above which clinical and virological protection (- - -, green shading) or clinical protection only (- · -, yellow shading) is expected. The horizontal bar shows the mean SRH value.

Antibodies to influenza A/equine/Richmond/1/2007 (H3N8) were detected in 592 of the 830 horses screened, giving an overall prevalence of 71.3% (95% CI: 68–74%) (Table 1). A mean SRH value of 78.2 mm^2^ (median = 88 mm^2^, interquartile range = 0–121 mm^2^) was obtained with a range of 0–208 mm^2^ (the lowest area measured was 12 mm^2^). Only 9% of horses had an SRH antibody value above 150 mm^2^; 44.5% horses had values between 85 and 150 mm^2^ and 46.5% had values less than 85 mm^2^ (Figure 3). 

There were significant differences in seropositivity in horses of different age, sex, breed and coat colour (Table 1). Although not statistically significant, the highest mean SRH values were seen in horses in Kaduna state (75.0%) and in horses used for polo (75.5%).

Univariable logistic regression showed that, compared to the young horses, adult and old horses had an OR of 1.6 (95% CI, 1.05–2.40) and 8.1 (95% CI, 2.75–24.1), respectively (Table 2). Male horses had an OR of 0.7 (95% CI, 0.52–0.99) compared to female horses. “Exotic” breeds of horses had an OR of 1.54, 95% CI: 1.06–2.22 compared to 1.00 and 0.96 for local and mixed breeds, respectively. Grey horses had an OR of 0.59 (0.39–0.90), which was significantly different (*p* = 0.015) to “basic”-coloured horses (the referent category) whereas the OR for “bright” coloured horses (0.86) was not significantly different. 

In the multivariate logistic regression analysis to identify confounding factors, only age remained a significant predictor of EIV seropositivity with an OR of 1.6 in adult horses and OR of 8.1 in old horses.

### 3.3. Seroprevalence of A/equine/Worcestershire/2019 (H3N8) among a Sample of Horses Positive for A/equine/Richmond/1/2007 (H3N8) in Northwest Nigeria

**Figure 4:**
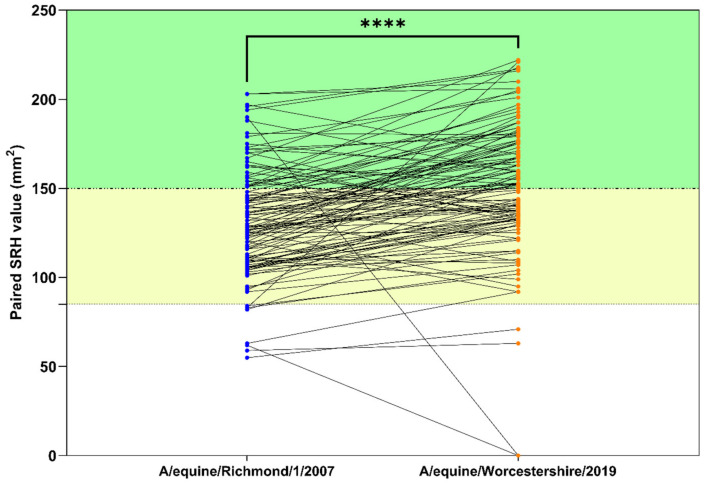
Comparison of single radial haemolysis antibody values measured against A/equine/Richmond/1/2007 or A/equine/Worcestershire/2019 among a subset of 118 horses in Northwest Nigeria. Dashed lines and shading indicate cut-off above which clinical and virological protection (- - -, green shading) or clinical protection only (- · -, yellow shading) is expected. **** indicates *p* = 0.0001 (Paired *t*-test).

The mean SRH value obtained with the 2019 virus (150.4 mm^2^) for 118 of the samples was significantly higher (*p* = 0.00001) than the mean value of 131.7 mm^2^ obtained with the 2007 virus (Figure 4). Of the 118 samples tested against both viruses, only 26.3% had SRH values at or above the level expected to provide clinical and virological protection when measured using the 2007 virus. Furthermore, 6.8% had SRH values below the cut-off expected to provide clinical protection. In contrast, around double the number of samples (52.5%) had SRH values above 150 mm^2^ when measured using the 2019 virus and only 3.4% had values below the lower threshold of 85 mm^2^.

## 4. Discussion

To the best of our knowledge, this is the first study to investigate the seroprevalence of EI among horses and the associated risk factors across states in Northwest Nigeria. Although EI virus was first isolated in tropical Africa in 1991 [17], earlier EI seroprevalence studies among horses in Nigeria focused only on polo horses during tournaments [22] and some selected stables in Kaduna State [24]. The results of this study therefore provide contemporary information that can be employed in steering the introduction of an effective action plan on the control of EI in Northwest Nigeria and the country at large. Nonetheless, donkeys might also contribute to the epidemiology of equine influenza as they represent around 80% of the equine population in Nigeria. Donkeys are predominantly found in northern Nigeria where they are used as work animals but as they are not bred locally (the majority are imported from the Republic of Niger [30]).

There have been growing concerns about the transboundary introduction and re-introduction of EI to Nigerian horses [19,20]. Sokoto State remains home to the old Sokoto caliphate with its boundaries including the present-day Burkina Faso, Cameroon, Niger and Nigeria. The border towns in Sokoto (Illela) and Katsina (Jibia and Daura) have large populations of horses. These border towns are arguably a gateway for movement of equids for trade and sports from Nigeria, through Niger to other West African countries. Kaduna and Kano are two states renowned for hosting colourful traditional durbars as well as local and international equine events in northern Nigeria. The overall seroprevalence of 71.3% (95% CI = 68.1–74.3) in this study corroborates the earlier finding of [18], which established that equine influenza exists among equids in all regions of Nigeria. However, the prevalence observed in this study is greater than previous reports from northern Nigeria [24], Brazil [31] and Spain [32]. The high prevalence observed might be due to a recent widespread exposure to equine influenza virus. Argentina has become an established source of equine importation into Nigeria [33], along with other sources like Europe and South Africa, due to the increasing interest in equestrian activities in Nigeria. The Argentine Ministry of Agriculture facilitated the export of 206 horses to Nigeria between 2018 and 2019. This probably explains the first report of an FC1 strain in some west African countries [19] as well as in West and Central African countries [20] coinciding with the 2019 outbreak of FC1 EI in South America (Argentina and Uruguay) [34]. Furthermore, with the rising cross-border equestrian activities, it is difficult to rule out neighbouring countries as sources of EI, as they are potential sources of equine importation to Nigeria. 

In univariable analyses, no significant association was found between location of horses and seropositivity, but polo horses had higher seropositivity and a significantly higher OR than horses used for other purposes. Polo horses might have had the highest EI seropositivity (75.5% (95% CI: 71–80)) because of the likelihood of being the most mobile compared to horses used for traditional durbar and mixed purposes. Although EI vaccines are not sold in Nigeria, vaccines are often imported [17], mostly along with importation of horses. The use of vaccines in the “exotic” breeds of horses, which includes 224 of the 331 horses involved in polo, might explain the higher seropositivity in these categories. The media reported that the 2018/2019 outbreak was followed by an EI vaccination campaign organized by the state government in July 2019, 6 months later, for a limited number of horses. 

Significantly more female horses were seropositive than males in univariate analysis. However, despite the higher seroprevalence observed in female horses, sex was not a predictor of EI seroprevalence in the multivariable analyses. This is consistent with a report in USA and Canada [35]. 

Grey coat colour was significantly associated with lower seropositivity and a lower OR in univariable analyses than other coat colours, but this significance was lost in the multivariable analyses. This may be due to the under-representation of grey-coat horses (13.2%) compared to the “basic”-coat-coloured horses among older horses resulting in lowering of study power to detect this effect as significant. The “bright” and grey coat colour categories were also over-represented among horses used for traditional durbars whereas polo ponies were more likely to be darker-coloured. 

Exploring the host factors associated with EIV seropositivity in both univariable and multivariable models, age was a consistently significant predictor of EI seropositivity. The significant progressive increase of EI seropositivity across age groups may be suggestive of recurring exposure in older horses. This finding agrees with studies from Brazil and Spain [30,31]. 

The mean SRH value obtained for all 830 horses with A/equine/Richmond/07 was only 78.2 mm^2^ (median = 88 mm^2^; interquartile range = 0–121 mm^2^) with 46.5% of horses having antibody levels below the value associated with clinical protection against a homologous virus challenge. A small proportion (9%) of the sampled equine population had antibodies likely to be fully protective against EI infection, similar to the finding in a report from Brazil [30]. The significantly higher mean SRH value obtained with a subset of 118 antibody-positive sera with A/equine/Worcestershire/2019 (H3N8) compared to A/equine/Richmond/1/07 (H3N8), which represents FC1 and FC2, respectively, is consistent with the recent circulation of an FC1 virus among horses in Nigeria. If the high seropositivity of EI among horses in Northwest Nigeria is indicative of higher rates of EI infection, then this could increase the risk of interspecies transmission of EI owing to the common practices of housing other domestic species in close proximity. These animals could play a role in the transmission dynamics and epidemiology of EI. In 2007, investigation of an EI outbreak in New South Wales inferred the possible role of birds as mechanical propagators of equine H3N8 virus [36]. The severe H3N8 EI outbreak that occurred in Jilin and Heilongjiang provinces of Northeast China in 1989 suggests the innate potential of influenza viruses to emerge in horses without reassortment [37]. The close interaction of various avian species and other mammals could result in novel influenza viruses like the avian-like A/equine/Jilin/1/89 (H3N8) in the future.

Keeping other animals like dogs, cats, ducks, ostriches, peacocks, sheep, goats, cattle, rabbits and pigeons in proximity with horses was commonly observed while sampling the horses in Northwest Nigeria. Bats were observed roosting in trees near where horses were tethered. Although influenza viruses have not been reported among bats in Nigeria, recent studies have demonstrated the abundance of some emerging viruses among bats sampled in Nigeria [38,39]. Since the first isolation of H3N8 equine influenza in dogs in 2004 [40], several reports have emerged of infection of dogs with equine influenza: a retrospective investigation in the UK disclosed the cause of the 2002 respiratory outbreaks among English foxhound to be an H3N8 virus of equine origin [41]. Just recently, some influenza A viruses including equine H3N8 were serologically detected in dogs in Poland [42]. Even though the equine/canine influenza lineages evolved independently from the avian H3 lineage and are unrelated to the human H3 lineage [43], the presence of other animals could provide opportunities for new interspecies transmission events.

## 5. Conclusions

Our study showed serological evidence for EI exposure in 71.3% of the tested population, with the majority of this presumed to result from infection rather than vaccination as vaccines are not routinely administered in Nigeria. The higher mean antibody values obtained using a more recent isolate in the SRH assay provides further evidence that the antibodies detected were likely due to infection during a recent outbreak rather than vaccination. Importantly, we also demonstrated an insufficient level of protection against EI in the region. Introduction of a vaccination programme with vaccines containing recently circulating FC virus, which could be targeted at horses that travel and intermingle, should mitigate against further outbreaks of equine influenza in Nigeria.

## Figures and Tables

**Table 1:** Factors associated with equine influenza seropositivity measured by single radial haemolysis assay using A/equine/Richmond/1/2007 (H3N8) virus among horses in Northwest Nigeria.

Factor	N	Positive	% Prevalence (95% CI)	*p*-Value ^1^
**AGE**				
Young (<5 years)	113	69	61.1 (52–70)	**0.0001**
Adult (5–14 years)	662	472	71.3 (68–75)	
Old (≥15 years)	55	51	92.7 (82–97)	
**SEX**				
Male	535	369	68.9 (65–73)	**0.043**
Female	295	223	75.6 (70–80)	
**BREED**				
Local	321	221	68.8 (64–74)	**0.033**
Exotic	268	207	77.2 (72–82)	
Mixed	241	164	68 (62–74)	
**LOCATION**				
Kaduna	256	192	75 (69–80)	0.443
Kano	314	221	70.4 (65–75)	
Katsina	122	85	69.7 (61–77)	
Sokoto	138	94	68.1 (60–75)	
**PURPOSE**				
Mixed	149	99	66.4 (58–74)	0.074
Polo	331	250	75.5 (71–80)	
Traditional Durbar	350	243	69.4 (64–74)	
**COAT COLOUR**				
Basic	640	469	73.3 (70–77)	**0.042**
Bright	80	55	68.8 (58–78)	
Grey	110	68	61.8 (52–70)	
**TOTAL**	**830**	**592**	**71.3 (68–74)**	

^1^*p*-values < 0.05 indicated in bold typeface, CI = 95% confidence interval, N = total number tested.

**Table 2:** Univariate logistic regression analyses of equine influenza virus seropositivity determined by single radial haemolysis assay with A/equine/Richmond/1/2007 (H3N8) among 830 horses in Northwest Nigeria.

FACTOR	N (n)	ODDS RATIO (95% CI)	*p*-Value ^1^
**AGE**			
Young (<5 years)	113 (69)	1.00	-
Adult (5–14 years)	662 (472)	1.58 (1.05–2.40)	**0.03**
Old (≥15 years)	55 (51)	8.13 (2.75–24.1)	**<0.0001**
**SEX**			
Female	295 (223)	1.00	-
Male	535 (369)	0.72 (0.52–0.99)	**0.04**
**BREED**			
Local	321 (221)	1.00	-
Exotic	268 (207)	1.54 (1.06–2.22)	**0.02**
Mixed	241 (164)	0.96 (0.67–1.38)	0.84
**LOCATION**			
Kaduna	256 (192)	1.00	-
Kano	314 (221)	0.79 (0.55–1.15)	0.22
Katsina	122 (85)	0.77 (0.47–1.24)	0.27
Sokoto	138 (94)	0.71 (0.45–1.12)	0.15
**PURPOSE**			
Mixed	149 (99)	1.00	-
Polo	331 (250)	1.56 (1.02–2.37)	**0.039**
Traditional Durbar	350 (243)	1.15 (0.76–1.73)	0.51
**COAT COLOUR**			
Basic	640 (469)	1.00	-
Bright	80 (55)	0.80 (0.48 – 1.33)	0.39
Grey	110 (68)	0.59 (0.39–0.90)	**0.015**
**TOTAL**	830 (592)		

^1^*p*-values < 0.05 indicated in bold typeface; CI = 95% confidence interval, N = total number tested; n = number positive

## Data Availability

Not applicable.
